# The Spinal Muscular Atrophy Disease Protein SMN Is Linked to the Golgi Network

**DOI:** 10.1371/journal.pone.0051826

**Published:** 2012-12-17

**Authors:** Chen-Hung Ting, Hsin-Lan Wen, Hui-Chun Liu, Hsiu-Mei Hsieh-Li, Hung Li, Sue Lin-Chao

**Affiliations:** 1 Institute of Molecular Biology, Academia Sinica, Taipei, Taiwan; 2 Graduate Institute of Life Sciences, National Defense Medical Center, Taipei, Taiwan; 3 Department of Life Science, National Taiwan Normal University, Taipei, Taiwan; Stanford University School of Medicine, United States of America

## Abstract

Proximal spinal muscular atrophy (SMA) is a neurodegenerative disorder caused by deficiency of the ubiquitous Survival of Motor Neuron (SMN) protein. SMN has been shown to be transported in granules along the axon and moved through cytoskeletal elements. However, the role and nature of SMN granules are still not well characterized. Here, using immunocytochemical methods and time-lapse studies we show that SMN granules colocalize with the Golgi apparatus in motor neuron-like NSC34 cells. Electron microscopy clearly revealed that SMN granules are transported into the Golgi stack and aggregate in the *trans*-Golgi apparatus. SMN granules are characterized as either coated or un-coated and behave like regulated secretory granules. Treatment of cells with monensin to disrupt Golgi-mediated granule secretion decreased SMN expression in neurites and caused growth cone defects similar to those seen in SMN knockdown cells. Knockdown of Cop-α, the protein that coats vesicles transporting proteins between the Golgi compartments, caused SMN granule accumulation in the Golgi apparatus. In addition to the well-studied role of SMN in small nuclear ribonucleoprotein (SnRNP) assembly, this work links SMN granules with the Golgi network and thus sheds light on Golgi-mediated SMN granule transport.

## Introduction

SMN protein deficiency causes human spinal muscular atrophy (SMA), which is characterized as an autosomal recessive neurodegenerative disorder [1]–[4]. SMN is produced by *SMN1* and its paralog gene, *SMN2*, which is nearly identical to *SMN1* with the exception of a C to T transition in exon 7 that causes alternative *SMN2* exon 7 splicing resulting in the exclusion of exon 7 in over 80% of *SMN2* transcripts and expression of an unstable SMN protein [5]–[7]. *SMN2* cannot compensate for the loss of *SMN1*; however, genetic evidence demonstrates that *SMN2* copy number influences disease severity, supporting the notion that *SMN2* could serve as a disease modifier [8]–[11].

SMN is ubiquitously expressed and is involved in snRNP assembly [12]–[14]. Additionally, SMN has been shown to move along the axon in granules [15], [16] and to form complexes that lack most of the interacting partners involved in snRNP assembly in motor neurons [17], [18]. Motor neurons lacking SMN show some axonal abnormalities [19]–[21] such as growth cone defects [19], suggesting a specific role of SMN granules in axons. SMN is known to interact with the α-coatomer (Cop-α) [22] that has been shown to mediate vesicle trafficking between the Golgi compartments [23]; however, a direct link between the SMN granule and the Golgi apparatus has not been shown.

Herein, we report that cytoplasmic SMN can be detected in the Golgi-enriched fractions. Time-lapse studies show that SMN granules associate with the Golgi apparatus and move like a regulated secretory granule. Global blockade of granule secretion from the *trans*-Golgi network resulted in decreased levels of SMN in neurites and a decrease in the size of growth cones. SMN granules retained in the Golgi apparatus were found in Cop-α knockdown cells, suggesting that Cop-α is involved in SMN granule secretion from the Golgi. Our work links SMN granules with the Golgi apparatus and implicates them in Golgi-mediated SMN granule transport.

## Materials and Methods

### Antibodies, Chemicals, Small Interfering RNAs, and Plasmids

Anti-Gemin2 (clone 2E17) was purchased from Sigma. Anti-SMN (clone 2B1) antibody was from BD Biosciences. Antibodies against Gm130 (clone EP892Y), Golgin-97, and Calnexin were from Abcam, and the antibody against Tgn38 was from Novus Biologicals. Anti-Syncrip and anti-COPα antibodies were from AVIVA Systems Biology. Antibodies specific for histone H1 (clone AE-4), α-tubulin (clone DM1A), β_III_-tubulin, and ChAT were purchased from Millipore. Anti-U2af65 and Alexa Fluor fluorescence dye-conjugated secondary antibodies (Phalloidin), as well as scrambled siRNA and siRNAs against mSmn (siRNA1∶5′-UAAAGUCAAUGGACGUAAUAGUAGC-3′; siRNA2∶5′-UACUAUUAGCUACUUCACAGGUCGG-3′; siRNA3∶5′-AAAUGUCAGAAUCAUCACUCUGGCC-3′; siRNA4∶5′-UGGCUAAGUGGUGUCGUCAUCAGCA-3′) were purchased from Invitrogen. Cop-α SMART-pool siRNAs and scrambled siRNAs were purchased from Thermo Scientific Dharmacon (5′-GGAACAAGCCCAACGCAAA-3′; 5′-GAACAGUACCUACGACCUA-3′; 5′-GGUUUGGGUUGCUCGGAAU-3′; 5′-CAAGUGAAGAUCUGGCGUA-3′). The pDsRed-monomer-β1,4-galactosyltransferase (β1,4-Gal-T) plasmid was purchased from Clontech. For constructing the mCherry-tagged SMAD anchor for receptor activation (SARA)-FYVE domain (mCherry-FYVE), the domain was amplified from pCMV5B-FLAG-SARA (Addgene plasmid 11738) [24] by PCR using a specific primer pair (forward: *Bgl* II-5′- ATCATTAGATCTGTGGCTCCAGTATGGGTACCGG-3′; reverse: *Xba* I -5′- TTAGATTCTAGATTACATTAGCACTGAATGGCAGATTACACAC-3′), and was then sub-cloned into the pGEM-T-Easy vector (Promega). The restriction enzymes *Bgl* II and *Xba* I were used to excise the cDNA fragment that was inserted into the pmCherry-C1 vector (Clontech) to generate mCherry-FYVE.

### Golgi Isolation

Golgi isolations were performed following the standard procedure provided with the Golgi isolation kit (Sigma). Briefly, about 19 dishes (10 cm^2^) of fresh NSC34 cells [25] at 90% confluency were washed with ice-cold PBS. Cells were then suspended in 1 mL of 0.25 M sucrose solution using 25G needles, and the cell suspension was sonicated. The homogenate was transferred to a centrifuge tube and centrifuged at 3,000× g for 15 min at 2–8°C. The supernatant was transferred into a fresh tube and the sucrose concentration in the sample (supernatant) was adjusted to 1.25 M by adding 2.3 M sucrose solution and mixing. Next, a discontinuous gradient was built in an ultracentrifuge tube. The order of sucrose gradient fractions in the tube (from bottom to top) was as follows: 1.84 M sucrose solution; sample (sucrose concentration adjusted to 1.25 M); 1.1 M sucrose solution; 0.25 M sucrose solution. The tubes were centrifuged at 120,000× g for 3 h at 2–8°C and the Golgi-enriched fraction was withdrawn from the 1.1 M/0.25 M sucrose interphase. Fractions were carefully isolated and analyzed by Western blot.

### Immunogold Labeling and Transmission Electron Microscopy Analysis

Cells were trypsinized, transferred to a carrier, and placed in an automatic freeze-substitution machine (Leica EM AFS2). Cells were freeze-substituted in acetone with 0.2% glutaraldehyde plus 0.1% uranyl acetate in ethanol at −90°C for 96 h. The temperature was elevated at a rate of 5°C/h to −60°C and samples were kept for 25.5 h. The temperature was then increased at a rate of 5°C/h to −20°C, followed by adding absolute ethanol. Samples were removed from the carrier, transferred to plastic capsules, and placed in a pre-cooled bottle filled with absolute ethanol at −20°C for 25.5 h. Samples were then infiltrated with LR gold resin in absolute ethanol [1∶1(v:v)] for 24 h and substituted for 112.5 h with three changes of pure LR gold. Non-catalyzed LR gold was preliminary activated with benzyl [100∶0.1(w:w)] for 24 h at −20°C with periodic mixing. Thereafter, samples were placed into gelatin capsules, covered with fresh resin, and polymerized by UV light at −20°C for 24 h. The temperature was elevated at a rate of 5°C/h to 25°C and samples were kept for 48 h. Thin sections (90 nm) were then prepared and blocked (5% normal goat serum in 50 mM Tris buffer, pH 7.4) for 1 h at room temperature (RT) and then incubated with anti-SMN antibody (1∶20) overnight at 4°C. After six washes (1 min each) in washing solution (1% normal goat serum in 50 mM Tris buffer, pH 7.4), samples were incubated with secondary antibody (12 nm gold-conjugated anti-mouse IgG/1∶50) for 0.5 h at RT. After six washes in washing solution and three dH_2_O washes (1 min each), samples were placed in 4% OsO_4_ for 10 min followed by twenty washes. After staining with 2% uranyl acetate for 2 min, samples were washed twenty times and stained with lead citrate for 8 min, followed by another twenty washes. For sciatic axons, samples were freshly isolated from mice and fixed (4% PFA, 0.25% glutaraldehyde in 0.1 M cacodylate buffer, pH 7.4) overnight. After three washes in buffer (0.1 M cacodylate buffer, pH 7.4) and two washes in dH_2_O (10 min each), samples were placed in 50% ethanol solution for 40 min and then washed twice with dH_2_O and buffer (10 min each). Samples were blocked (2% BSA, 10% normal goat serum in 0.1 M cacodylate buffer, pH 7.4) for 1 h and then incubated with anti-SMN antibody (1∶50) for 2 days at 4°C. After two washes in blocking solution (10 min each) followed by two washes in washing solution (8% BSA, 3% normal goat serum in 0.1 M cacodylate buffer, pH 7.4) (30 min each), samples were incubated with secondary antibody (1.4 nm gold-conjugated anti-mouse IgG, 1∶100) overnight at 4°C. After two washes in washing solution and buffer washes (10 min each), samples were placed in 2% glutaraldehyde (in 0.1 M cacodylate buffer, pH 7.4) for 15 min and washed in cacodylate buffer two more times (10 min each) and then in 0.2 M sodium citrate buffer five times (1 min each). Silver enhancement (Nanoprobe) was then performed for 10 min, followed by five washes in 0.2 M sodium citrate buffer (1 min each) and two washes in buffer (10 min each). Samples were then fixed in 1% OsO_4_ in 0.1 M cacodylate buffer for 1 h, washed twice with dH_2_O (10 min each), and finally subjected to dehydration, infiltration, embedding, and sectioning.

### Cell Culture and Knockdown Assay

Materials used for cell culture were purchased from Invitrogen. Murine NSC34 cells were cultured in DMEM with 10% fetal bovine serum (FBS, Hyclone) and 1% penicillin and streptomycin as previously described [25]. Differentiated NSC34 (NSC34D) cells were cultured according to a previous report [26]. For knockdown assays, 7.5×10^4^ cells were seeded on a poly-L-lysine-coated 18 mm micro cover glass (Matsunami) in a 12-well plate. After overnight culture, scrambled siRNA and siRNAs specific against mSmn (siRNA3) were transfected using RNAiMAX reagent (Invitrogen) following the manufacturer’s instructions. Cells were knocked down for 48 h and then harvested for Western blot or immunocytochemical studies. For plasmid DNA transfection, PolyJet reagent (SignaGen Laboratories) was used following the manufacturer’s protocol.

### Time-lapse Studies

To perform time-lapse studies, 2×10^5^ NSC34D cells were first seeded on poly-L-lysine (Sigma, 100 µg/ml) coated glass slides in 6-well culture plates. To visualize SMN granules and SMN granules associated with the Golgi, EGFP-tagged SMN (EGFP-SMN) and dsRed monomer-tagged β1,4-Gal-T (dsRed-β1,4-Gal-T) were transiently transfected (or co-transfected) into cells. For the control experiment, SMN-EGFP was co-transfected with the mCherry-FYVE plasmid. After 24 h, a glass slide was placed into a Perfusion, Open, and Closed (POC) chamber (Pecon) and placed into a deconvolution microscope (Olympus IX-71)-attached incubator (DeltaVision Core, Applied Precision, LLC) (37°C for 30 minutes). Images, including *z* stacks, were recorded under a 60× objective lens (Olympus PlanApo 60×, N.A. 1.42, WD 0.15) at indicated time intervals (every 5 seconds or every 10 seconds), using the GFP (EX 470/40, EM 525/50 for EGFP) and mCherry (EX 572/35, EM 632/60) filter sets. Images were taken and deconvolution analysis was conducted using the integrated SoftWoRx imaging program. Time-series images (including *z* stacks) for colocalization studies were not projected. The real-time three-dimensional colocalization of SMN and organelle markers in a field of view was determined and confirmed using Imaris software (v.7.0.0, Bitplane).

### Immunocytochemistry and Confocal Microscopy Analysis

Cells were washed with pre-warmed 1× PBS (37°C) and fixed with 4% paraformaldehyde for 15 min at room temperature (RT). Cells were then permeablized in 1× PBS containing 0.1% Triton-X-100 at RT for 12 min. After 3 washes, cells were blocked in blocking solution (PBS containing 0.1% Tween 20 with 5% BSA) for 1 h at RT, followed by incubation with primary antibodies in blocking solution for 3 h at RT. After three washes, cells were incubated with appropriate Alexa Fluor 488/555/647-conjugated secondary antibodies in blocking solution for 1 h at RT. Cells were washed with 1× PBS 3 times and mounted using Prolong mounting reagent (Invitrogen). Images (including *z* stacks) were observed with a 63× objective (Carl Zeiss Plan-Apochromat 63×, N.A. 1.4 Oil DIC), digitally acquired using the LSM 510 Meta confocal microscope (Carl Zeiss), and processed using MetaMorph software (v7.7.2). The colocalization of mSmn granules with the Golgi markers (*cis*-Golgi marker: Gm130; *trans*-Golgi markers: Tgn38, Golgin-97, and WGA) in a selected Golgi-enriched area was measured using a plugin in MetaMorph software. To determine the transfection efficiency ( =  number of cells expressing any fluorescent protein/all cells) of the cotransfection assays, over sixteen different fields were counted from four independent transfections (over 4800 cells were counted in each group). All images were observed with a 20× objective (Carl Zeiss FLUAR 20×, N.A. 0.75), digitally acquired using an LSM 510 Meta confocal microscope (Carl Zeiss), and processed using LSM image examiner software (Carl Zeiss).

### Isolation of Neurite Fraction

The NSC34D cell neurite fraction was isolated following the protocol provided in the Neurite Outgrowth Quantification Assay Kit (Millipore), with some modifications. Briefly, 2×10^5^ cells were seeded on the transwell insert (polycarbonate membrane with 3 µm pore size and pre-coated with poly-L-lysine) in a 6-well plate (Corning). Cells were incubated for 24 h in differentiation medium and then treated with either ethanol or monensin (5 µM, in absolute ethanol solution) for 6 h. The cell bodies from the upper surface of the membrane were removed with a cotton swab and neurite fractions were extracted for Western blot analysis.

### Western Blot Analysis

Cells and neurite fractions from either ethanol-treated or monensin-treated groups were harvested and lysed, and Western blotting was performed as previously described (20). Antibody dilutions were as follows: Cop-α, 1∶1000; SMN, 1∶5000; Calnexin, 1∶1,000; Gm130, 1∶5000; Tgn38, 1∶500; Syncrip, 1∶1,000; Pabp-C1, 1∶5,000; Gemin2, 1∶2000; ChAT, 1∶500; U2af65, 1∶10,000; histone H1, 1∶1000; α-tubulin, 1∶10,000; β_III_-tubulin, 1∶10,000; HRP-conjugated secondary antibodies, 1∶5000. Western blot quantification was performed by scanning the autoradiographs with a computerized densitometer. Signal intensities were determined by densitometric analysis (AlphaInnotech) using the AlphaImager program. The results expressed as relative densitometric units and calculated as the ratio between the densitometric units of each protein band in each fraction divided by the densitometric units of fraction 5, were analyzed by the same program.

### Statistical Analysis

Experiments were performed in triplicate. Data were analyzed by Prism 5 software (GraphPad). The statistical significance of any difference between group means was determined by applying a two-tailed *t*-test. The difference was considered statistically significant if *P* < 0.05.

## Results

### SMN Granules Colocalize with the Golgi Network

**Figure 1:**
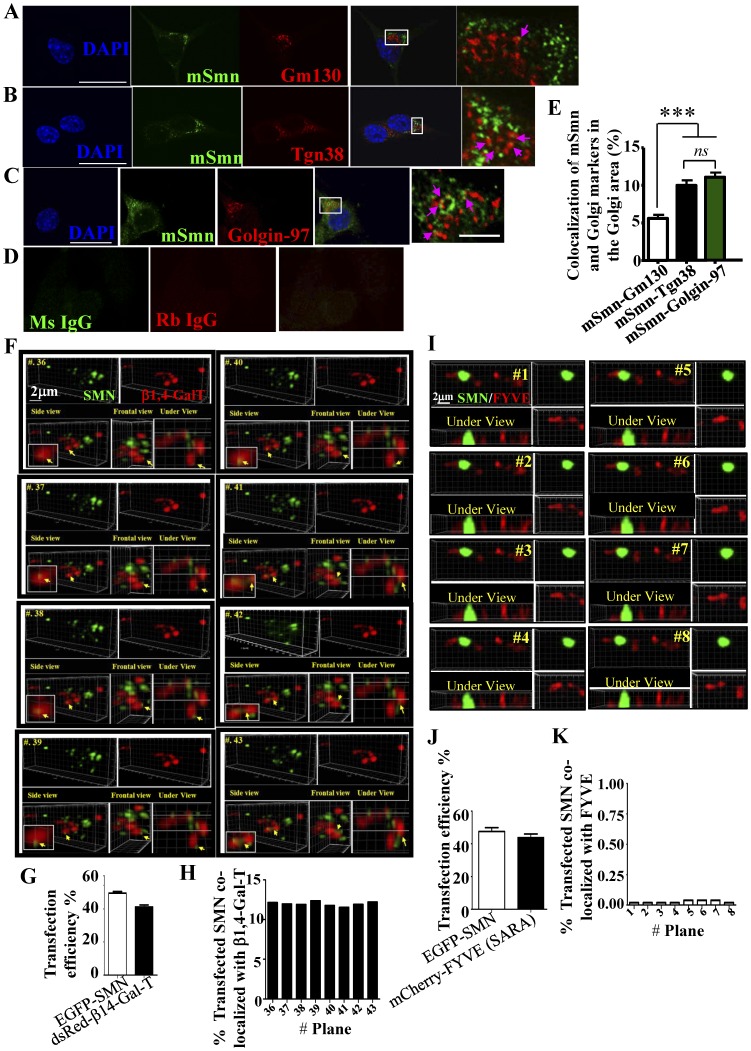
Colocalization of SMN with the Golgi apparatus. (A–C) NSC34D cells were stained with SMN antibody (green) and antibodies against Gm130 (A), Tgn38 (B), and Golgin-97 (C) (red). Magnified regions are shown in boxes at right of the merged figures. Obvious colocalization between each protein is indicated with pink arrows. Scale bar: 20 µm; 2 µm in magnified box. (D) In the control group, antibodies against mouse (Ms) or rabbit (Rb) immunoglobulin G (IgG) were used in place of SMN antibody or the Golgi marker antibodies. (E) Quantitation of the colocalization ratio between SMN with Gm130, Tgn38, Golgin-97, and WGA in selected Golgi enriched region. Data represent the mean ± SEM from three independent experiments (over 50 cells were counted in each group). ***, *P<*0.001. *ns*: not significant. (F) EGFP-SMN and dsRed-β1,4-Gal-T or (I) EGFP-SMN and mCherry-FYVE are shown in green and red, respectively. Time series images were taken every 10 sec, including *z*-stacks. Images are shown in three dimensions with views from the front, underside, and side-back (F). Selected planes 36–43 (F) and 1–8 (I) are shown. Arrows indicate obvious colocalizations of SMN and the Golgi. (G, J) Transfection efficiencies of EGFP-SMN with dsRed-β1,4-Gal-T were 49.4% ±1.0% with 41.1% ±1.2%, respectively; efficiencies for EGFP-SMN with mCherry-FYVE were 47.5% ±2.3% with 43.8% ±2.2%, respectively. Data represent the mean ± SEM, *n* = 4 in each group. (H, K) Quantitation of the colocalization ratio in each time-point between SMN with organelle markers in a selected view from a neurite. Bar: 2 µm.

To investigate the interaction between the murine Smn (mSmn) granules and the Golgi network, NSC34D cells were stained with antibodies against SMN (green), Gm130 (*cis*-Golgi marker protein, red) (Figure 1A), Tgn38 (Figure 1B), Golgin-97 (Figure 1C) (*trans*-Golgi markers, red), and fluorescent signals were observed by immunofluorescence microscopy. The results indicate that mSmn granules colocalize with the Golgi apparatus (Figure 1A–C, see magnified box, indicated with arrows) and more mSmn granules were found to colocalize with the *trans*-Golgi apparatus (10.7±0.7% determined by Tgn38 staining and 10.1±0.6% determined by Golgin-97 staining) than the *cis*-Golgi apparatus (6.1±0.5% determined by Gm130 staining) at the Golgi enriched foci (*n* > 50 cells in each group, *P* < 0.001) (Figure 1E). To directly evaluate the connection between SMN granules and the Golgi apparatus, EGFP-SMN and dsRed-β1,4-Gal-T (*trans*-Golgi marker) were cotransfected into NSC34D cells and their colocalization in neurites was evaluated every 10 sec for 15 min. We studied their colocalization in neurite because cytoplasmic SMN may dominantly associate with the snRNP assembling complex. In addition, the *trans*-Golgi apparatus is known to localize to outposts in neurite [27]. The dynamics of both β1,4-Gal-T and the SMN granules were recorded by deconvolution microscopy. To prevent protein-protein colocalization artifacts, data are presented in three different views (side, frontal, and bottom views) and were analyzed in three-dimensions through built-in Imaris software (v.7.0.0) [Selected planes (#36–43) are shown in Figure 1F, colocalization is indicated by arrows; full movie can be found in Movie S1]. The approximate transfection efficiency was 41% for the dsRed-β1,4-Gal-T and 49% for the EGFP-SMN (Figure 1G). About 11–12% of transfected SMN granules colocalized with the *trans*-Golgi outposts were found in the selected view from the neurite (Figures 1F, side view, H). To rule out the possibility that the colocalization was caused by forced overexpression of the plasmids, EGFP-SMN was cotransfected with mCherry-FYVE, which targets to the endosomal membrane (Figure 1I). The approximate transfection efficiency was 43% for the mCherry-FYVE and 48% for the EGFP-SMN (Figure 1J). Time-lapse study followed by colocalization analysis showed that EGFP-SMN and mCherry-FYVE have a very low colocalization ratio (Figure 1K) [Selected planes (#1–8) are shown in figure 1I and full movie can be found in Movie S2]. These results demonstrate that SMN granules colocalize dynamically with the *trans*-Golgi outpost.

### SMN Granules Exist in the Golgi Enriched Fractions

**Figure 2:**
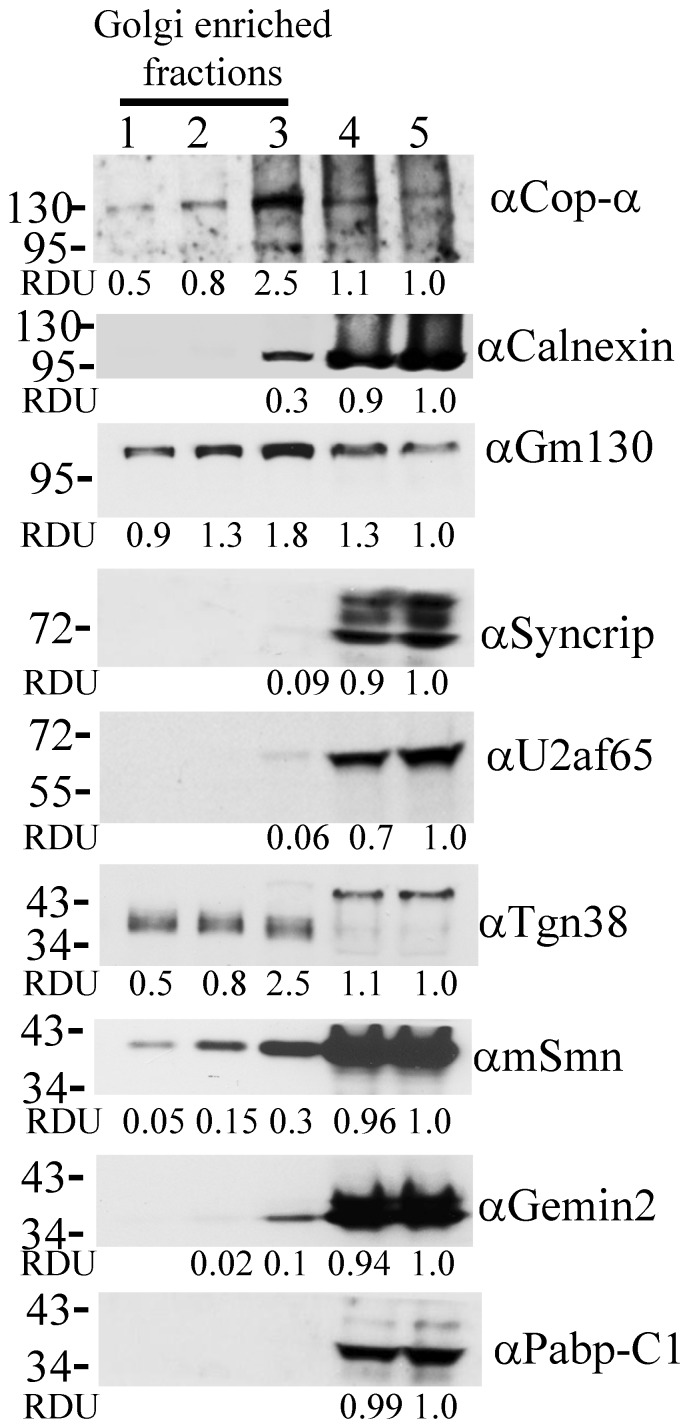
Cytoplasmic mSmn exists in the Golgi-enriched fractions. Protein lysates including Golgi-enriched fractions (lanes 1–3) and other fractions (lanes 4–5) were analyzed by Western blotting with Cop-α, Gm130, Calnexin, Syncrip, U2af65, Tgn38, SMN, Gemin2, Pabp-C1, and VAPB antibodies. The relative amounts of proteins in each lane are expressed in relative densitometric units (RDUs).

To substantiate this finding, Golgi fractions were isolated from NSC34 cells and blotted with the anti-Golgi antibodies Gm130 and Tgn 38 (Figure 2, lane 1–3). The protein amount in each fraction is relative to fraction 5 and is presented in relative densitometric units (RDU). The mSmn signal was detectable in all Golgi-enriched fractions (Figure 2, lanes 1–3). However, the amounts of mSmn in Golgi-enriched fractions were relatively less when compared to the other fractions (Figure 2, lane 4–5). Further, we examined known SMN-associating partners [Cop-α, Syncrip, poly(A) binding protein (Pabp) C1, and Gemin2] to determine whether they also localized in the Golgi-enriched fractions. Cop-α has been shown to move with mSmn granules along the axon [22]. Gemin2 tightly associates with SMN to form the cytoplasmic SMN complex to mediate snRNP assembly [12], [13]. Syncrip and Pabp-C1 are associated with Smn granules and have been suggested to be involved in axonal mRNA transport [19], [28], [29]. Only Cop-α, but not Pabp-C1, Gemin2, or Syncrip, had a similar staining pattern to mSmn, suggesting that Cop-α, a protein involved in coating vesicles during secretion from the Golgi apparatus, may be involved in mSmn granule secretion. An ER marker protein, calnexin, and splicing factor U2af 65 kDa subunit (U2af65) served as negative controls. These results indicate that a small portion of cytoplasmic mSmn exists in the Golgi fraction and suggest that Cop-α may be involved in mSmn granule secretion from the Golgi network.

### SMN Granules Move Through the Golgi and Act Like Regulated Secretory Granules

**Figure 3:**
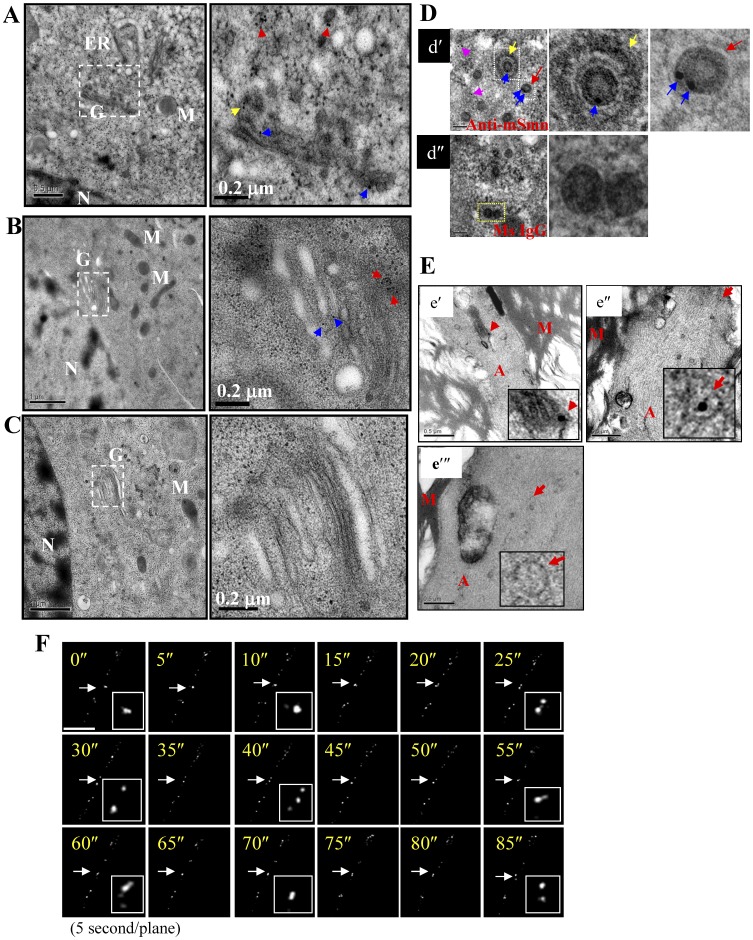
Characterization of mSmn granules through immuno-gold labeling TEM. (A–C) Electromicroscopic analysis of NSC34 cells revealed mSmn signals can be found in the Golgi stack (indicated by blue arrows) and mSmn aggregates at the *trans*-Golgi membrane (indicated by a yellow arrow). Magnified images from the boxed areas show the mSmn signal in the Golgi secreted granules (indicated by red arrows). Bar: 0.5 µm (left) and 0.2 µm (right). In the control group (C), mouse IgG was used in place of SMN antibody. Bar: 0.5 µm (A, left), 1 µm (B, C, left) and 0.2 µm (right). N, Nucleus; G, Golgi; M, Mitochondria; ER, Endoplasmic Reticulum. (D) Electromicroscopic analysis characterizes different mSmn granules (d′, blue arrows indicate mSmn granules; red arrows indicate non-granule type mSmn signals in the cytoplasm). Note that some mSmn granules were coated (d′, middle) and some were uncoated (d′, right). In the control group, mouse IgG instead of SMN antibody was used (d″). Bar: 100 nm. (E) Electromicroscopic analysis of sciatic axons revealed that the mSmn signal presents in granules and in the axoplasm. M, myelin sheath; A, axoplasm. The mSmn signals are indicated with red arrows. Figure E–e′ shows an mSmn granule (red arrow) that appears in a long sausage-shaped organelle. Figure E–e″ shows a small mSmn granule (red arrow) in an axon. Figure E–e′″ shows the control group, using the mouse IgG instead of SMN antibody. Bar: 0.5 µm. (F) Time series images (5 sec/plane) acquired from EGFP-SMN-transfected NSC34D cells show constant fusion and fission of SMN granules in the axon (indicated with an arrow). Bar: 20 µm.

To further characterize the nature of mSmn granules, transmission electron microscopy (TEM) analysis was performed to examine the ultrastructure of mSmn granules in the NSC34 cells (Figure 3A–D) and sciatic axons (Figure 3E). Immuno-gold labeling revealed mSmn signals in the Golgi stacks (Figure 3A, B, indicated by blue arrows shown in the right magnified boxes), aggregating in the *trans*-Golgi apparatus (Figure 3A, indicated by a yellow arrow shown in the right magnified box) or secreted granules (Figure 3A, B, indicated by red arrows shown in the right magnified boxes), indicating that mSmn granules are secreted through the *trans*-Golgi network. However, the gold positive mSmn signal within the Golgi was less abundant throughout the cell (data not shown). This data is consistent with previous data acquired from Western blot analysis, showing that less mSmn is detectable in the Golgi-enriched fractions (Figure 2, lanes 1–3) when compared to other fractions (Figure 2, lanes 4–5). In addition, electromicroscopic studies also show that both coated (Figure 3D–d′, left and middle, indicated with a yellow arrow) and uncoated mSmn granules (Figure 3D–d′, left and right, indicated by a red arrow) could be observed under high magnification (Figure 3D–d′, mSmn signals are indicated with blue arrows). In addition, numerous mSmn-positive signals were detected apart from the granules (Figure 3D–d′, left, indicated with pink arrows), representing other cytoplasmic mSmn-associated complexes. In addition, sciatic nerves of adult mice were isolated and examined for mSmn granules. The mSmn signal was found in an unidentifiable organelle (Figure 3E–e′, indicated with a red arrow) and in the granule (Figure 3E–e″, indicated with a red arrow).

Regulated secretory granule biogenesis requires a number of maturation steps. These include the sorting of in/out proteins, the removal of the clathrin coat and other coat proteins from the granule, the removal of water, and the condensation of granule contents to form mature granules [30]. The observation that some mSmn granules were coated while some were uncoated led us to hypothesize that the mSmn granule may be processed like some regulated secretory granules [30]. To confirm this possibility, the motion of individual SMN granules (tagged with EGFP) in NSC34D cells was examined using real-time deconvolution microscopy analysis at 5 sec intervals. The pattern revealed that the granules are likely undergoing constant assembly and disassembly in neurites (Figure 3F). Together, these results support that mSmn granules behave like regulated secretory granules.

### Global Disruption of Golgi-mediated Granule Secretion Decreased SMN Levels in Neurite

**Figure 4:**
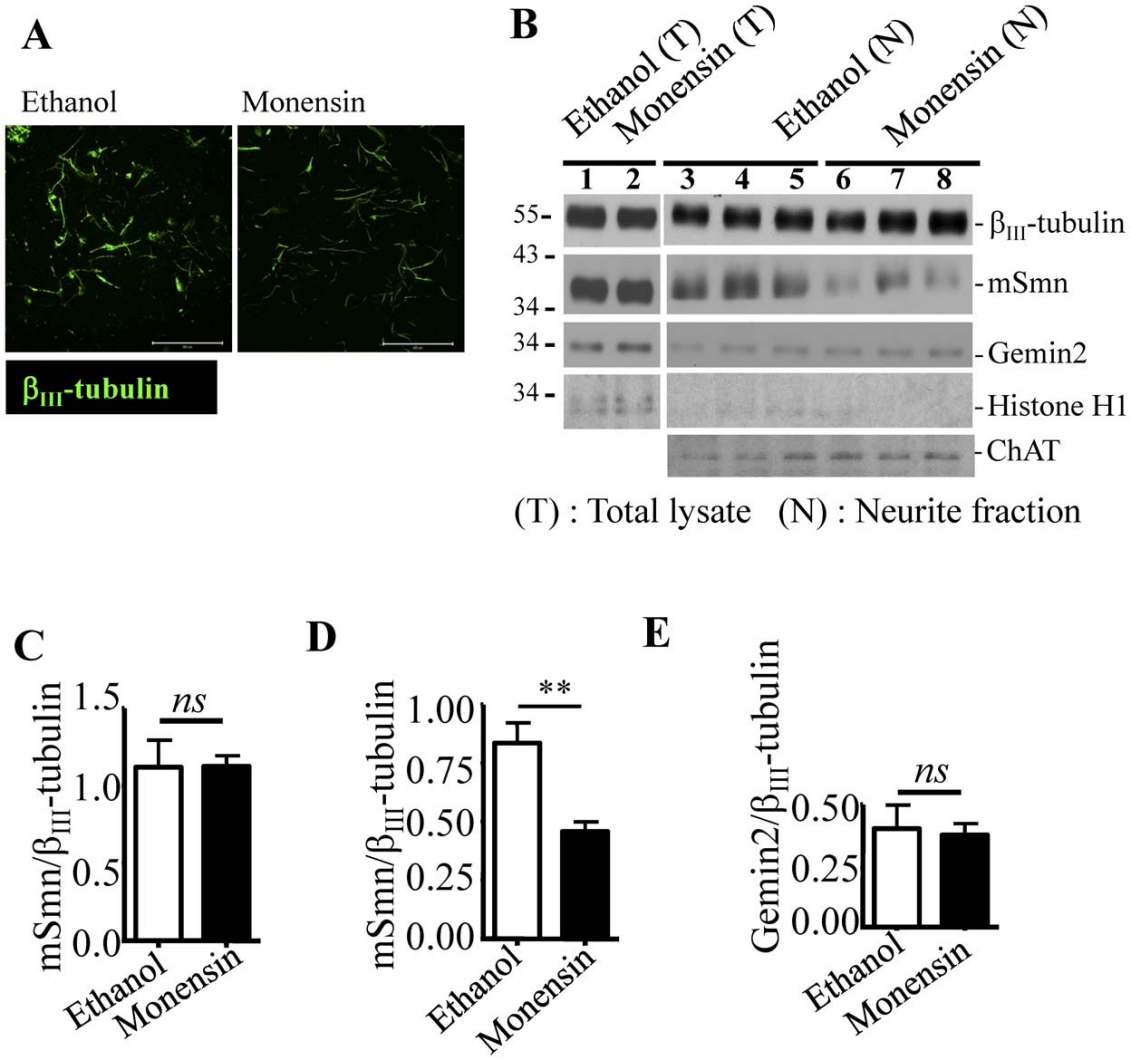
Global disruption of the *trans*-Golgi mediated granule secretion reduces mSmn levels in neurites. (A) The underside of the polycarbonate membrane that allowed neurite outgrowth was stained by β_III_-tubulin antibody (green). Bar: 200 µm. (B) Total lysates (T, lanes 1–2) and neurite lysates (N, lanes 3–8) (lanes 3–5: ethanol treated control; lanes 6–8: monensin treated cells) were analyzed for β_III_-tubulin (internal control), mSmn, Gemin2, histone H1, and ChAT expression by Western blotting. Histone H1 served as a negative control for the neurite fraction. (C–E) Quantitative analysis of the blot shown in panel A using β_III_-tubulin for normalization. mSmn levels in the total lysate (C) and neurite fraction (D), and Gemin2 level (E) in the neurite fraction in the two treatment groups. Results represent the mean ± SEM from at least three independent experiments performed in triplicate. *ns*: not significant; **, *P* < 0.01.

Since mSmn granules are linked with the Golgi apparatus, we asked whether disruption of Golgi-mediated granule secretion would affect mSmn level in neurites. The Na^+^ ionophore antibiotic monensin was used to disrupt the *trans*-Golgi membrane and block granule secretion in NSC34D cells. Cells seeded on a transwell (with a polycarbonate membrane) allowed us to isolate neurite fractions from the underside of the membrane (Figure 4A, neurites are stained by β_III_-tubulin and shown in green). Neurite fractions from cells treated with either ethanol (solvent mock control) or monensin were extracted and analyzed by Western blot. There was no significant difference in mSmn levels in total cell lysates from either ethanol or monensin treated groups (Figure 4B, lanes 1–2) (1.12±0.18 in the ethanol-treated group *vs.* 1.13±0.07 in the monensin-treated group, *n* = 3, Figure 4C). However, the mSmn levels in the neurite fractions of monensin-treated groups (Figure 4B, lane 6–8) were significantly lower than those in ethanol-treated groups (Figure 4B, lane 3–5) (0.46±0.04 in the monensin-treated group *vs*. 0.84±0.09 in the ethanol-treated group) (Figure 4D, *n* = 9). In contrast, no significant differences were observed in Gemin2 levels [0.38±0.05 in the monensin-treated group (Figure 4B, lanes 6–8) *vs*. 0.40±0.10 in the ethanol-treated group (Figure 4B, lanes 3–5)] (Figure 4E), indicating that Gemin2 was not affected when Golgi-mediated granule secretion was blocked. These findings indicate that disruption of the Golgi may influence mSmn granule transport.

### Global Disruption of Golgi-mediated Granule Secretion Causes Smaller Growth Cone Size

**Figure 5:**
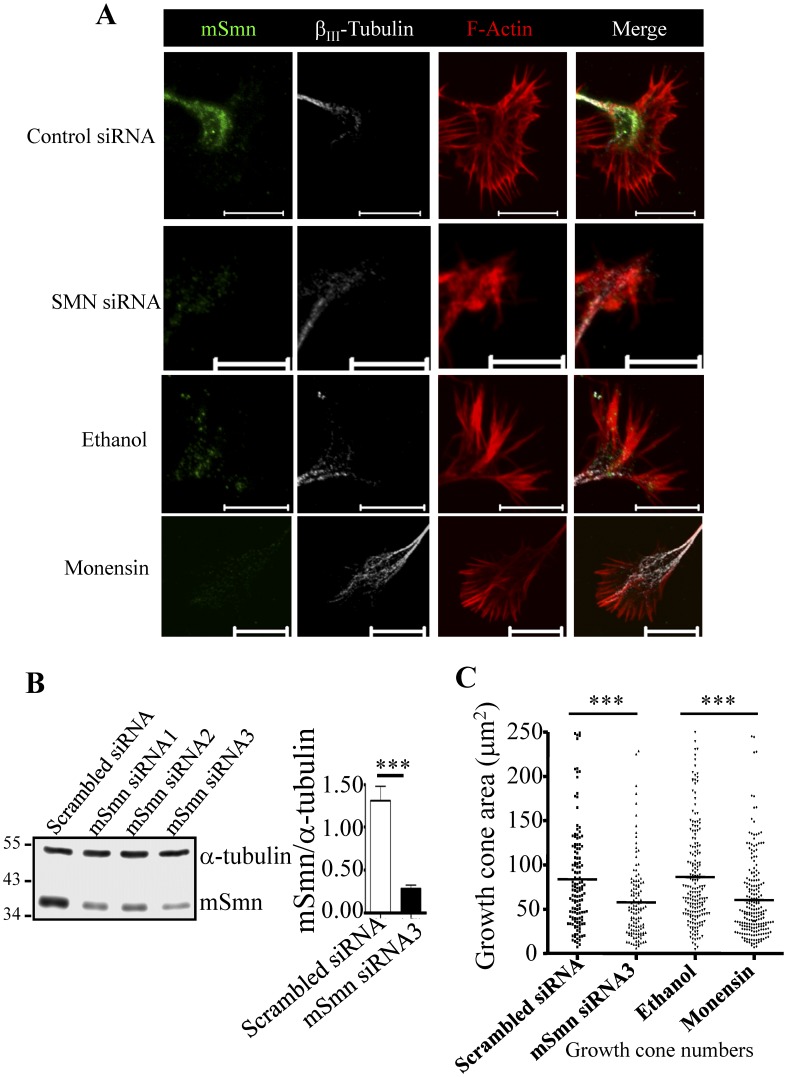
Global disruption of the *trans*-Golgi mediated granule secretion mimicked SMN-deficient growth cone defects. (A) Growth cones in NSC34D cells receiving different treatments were visualized by staining with SMN antibody (green), β_III_-tubulin antibody (white), and Alexa Fluor 647-conjugated phalloidin (red). Bar: 10 µm. (B) Analysis of mSmn knockdown by three siRNAs specific against Smn and quantitation of the knockdown of Smn by siRNA3, the siRNA achieving the highest efficiency. Data represent the mean ± SEM from at least three independent experiments performed in triplicate. ***, *P<*0.001. (C) Growth cone area of NSC34D cells treated either with scrambled siRNA (*n* = 133) or mSmn siRNA-3 (*n* = 120) and ethanol (*n* = 188) or monensin (*n* = 207) were quantitatively analyzed. ***, *P* < 0.001.

Because global inhibition of granule secretion decreased mSmn levels in neurites, we further investigated whether this may lead to similar defects caused by SMN deficiency in NSC34D cells. SMN granules have been implicated in β-actin transport [16]–[19]. β-Actin is enriched in the growth cone and therefore mSmn-deficient motor neurons may be expected to exhibit defects in β-actin transport and cause growth cone defects [19]. The growth cone was examined by staining for F-actin (red) and β_III_-tubulin (white) followed by confocal microscopy (Figure 5A). Three mSmn-specific siRNAs (targeting the mSmn coding regions) were used to knock down mSmn in cells. Cells treated with mSmn siRNA3 led to a 76% reduction in the amount of cellular mSmn (0.28±0.04 in the mSmn siRNA3-treated group *vs.* 1.31±0.17 in the scrambled siRNA-treated group, *n* = 4) (Figure 5B). The average growth cone size in the group treated with mSmn siRNA3 was significantly smaller than that of the scrambled siRNA-treated group (57.81±4.03 *vs.* 83.78±4.96 µm^2^, *n* = 133 *vs.* 120) (Figure 5C, left). This result is consistent with previous work indicating that SMN-deficient motor neurons showed growth cone defects [19]. Furthermore, cells treated with monensin to disrupt Golgi-mediated granule secretion also showed a smaller growth cone size (60.57±3.15 µm^2^, *n* = 207) as compared to the ethanol-treated group (86.51±4.03 µm^2^, *n* = 188) (Figure 5C, right). These results demonstrate that global blockage of Golgi-mediated granule secretion causes growth cone defects similar to those caused by mSmn knockdown in NSC34D cells.

### Cop-α is Involved in mSmn Granule Secretion from the Golgi

**Figure 6:**
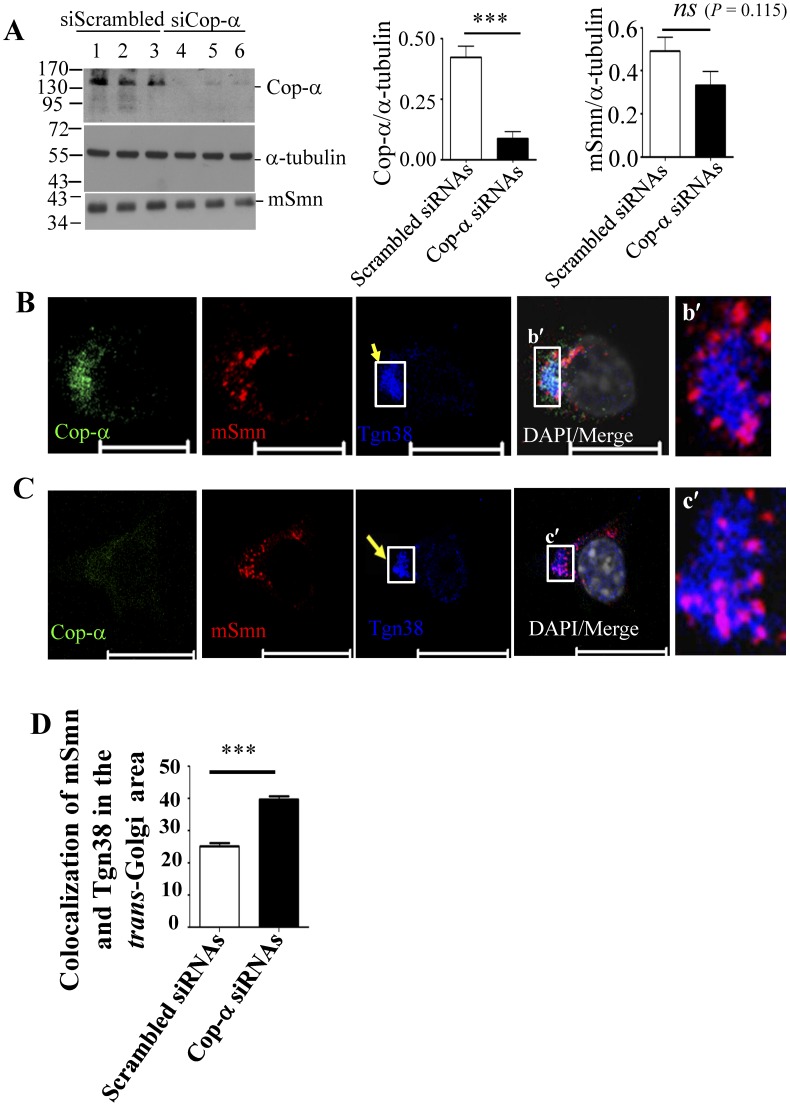
Cop-α is involved in mSmn granule secretion from the Golgi apparatus. (A) Cell lysates treated with scrambled siRNA (lanes 1–3) or Cop-α siRNAs (lanes 4–6) were analyzed for α-tubulin (internal control) and Cop-α expression by Western blotting. Quantitation of the blot using α-tubulin for normalization of Cop-α and mSmn expression level in the total lysates. Results represent the mean ± SEM from at least two independent experiments performed in triplicate. ***, *P* < 0.001. *ns*: not significant. (B, C) Immunocytochemical analysis of the mSmn granules and Tgn38 colocalization in proximity to the Golgi in NSC34D cells treated with scrambled siRNA (B) or Cop-α siRNA (C). Cells were stained with antibodies against Cop-α (green), mSmn (red), and Tgn38 (blue). DAPI was used for nuclei staining (white, shown in the merged panel). Bar: 10 µm (B); 20 µm (C). The boxed areas (b′ and c′) in the merged panel show the *trans*-Golgi area (for quantitation of the colocalization) and are shown magnified at the right side. Yellow arrows in (B) and (C) indicate the colocalization of mSmn granules with the *trans*-Golgi area. (D) Quantitative analysis of the colocalization of mSmn granules with Tgn38 in NSC34D cells treated either with scrambled siRNAs or Cop-α siRNAs. Results represent the mean ± SEM from at least two independent experiments performed in triplicate. ***, *P* < 0.001.

Cop-α was recently confirmed as an SMN interacting protein and is known to coat granule transporting proteins in the Golgi membrane and move with mSmn granules along the axon [22]. We asked whether Cop-α is possibly involved in mSmn granule secretion from the Golgi apparatus. Cop-α siRNAs were used to knock down Cop-α and led to an 80% reduction in Cop-α expression (0.42±0.05 in the scrambled siRNA-treated group *vs.* 0.09±0.03 in the Cop-α siRNA-treated group, *n* = 6) in NSC34D cells (Figure 6A). The amount of mSmn was not significantly affected by Cop-α knockdown (0.49±0.06 in the scrambled siRNA-treated group *vs.* 0.33±0.06 in the Cop-α siRNA-treated group, *n* = 6) (Figure 6A). To analyze Cop-α involvement in mSmn granule secretion from the Golgi, the co-localization of mSmn granules with the *trans*-Golgi apparatus was further evaluated after Cop-α knockdown through confocal microscopy. Cells transfected with scrambled siRNA showed about 25.06±1.13% of mSmn co-localized with Tgn38 (*n* = 104) in the *trans*-Golgi area (Figures 6B–b′, D); however, significantly more mSmn granules colocalized with Tgn38 (39.63±1.04%, *n* = 122) in Cop-α siRNA treated cells (Figure 6C–c′, D), indicating that mSmn granules accumulated in the *trans*-Golgi apparatus. These results show that Cop-α is involved in mSmn granule secretion from the *trans*-Golgi membrane.

## Discussion

### Cytoplasmic SMN Granules Move Through the Golgi Network

As SMN moves as granules, it is hypothesized to have specific functions. Recently, the Golgi-associated vesicle coat complex Cop-α was shown to associate and migrate together with SMN granules within axons [22]. Here we found that Cop-α knockdown led to SMN granule accumulation in the Golgi apparatus, indicating that Cop-α is involved in mSmn granule secretion from the Golgi. Interestingly, SMN granules seem to constantly assemble and disassemble (Figure 3D) within neurite processes. This may be because freshly secreted SMN granules are premature and need to undergo further homotypic fusions, in a manner similar to regulated secretory granules. Freshly secreted, coated regulated granules continuously fuse and split to remove their coat and condense their cargos [30]; we found that both “coated” and “uncoated” mSmn granules exist in NSC34 cells (Figure 3C–c′), further strengthening the possibility that some sort of condensation process is required for mSmn granule maturation. A previous report also indicated that the dynamic dissociation and re-association of SMN with α-COP granules implied that SMN is not contained within a coated vesicle [22]. However the SMN signals are found in both the “coated” and “uncoated” granule (Figure 3B) and in the Golgi stacks (Figure 3A, B), indicating that SMN is possibly carried as cargo in Cop-α associated vesicles. SMN granules have been reported to associate with different cellular proteins, such as Gemin2/Gemin3 [12], [13], [17], [18] and RNA-binding proteins/mRNAs [18], [28], [31]–[33]. However, blocking granule secretion, while reducing mSmn levels, did not affect Gemin2 levels in the neurite fraction (Figure 4B), suggesting that Gemin2 is not transported through a Golgi-mediated secretion pathway. In addition, Syncrip and Pabp-C1 are known to associate with Smn granules and have been suggested to be involved in axonal mRNA transport [19], [28], [29], but we found different localization patterns with Smn in Golgi-enriched fraction (Figure 2), indicating that those proteins, like Gemin2, are not transported through the Golgi secretion pathway. Several reports have indicated that less than 50% of SMN granules colocalize with Gemin2/Gemin3 [17], [18] or RNA-binding proteins (Syncrip/Pabp-C1) [18], [28], [31]–[33], suggesting that they colocalize with each other in a dynamic motion. Another possibility is that multiple-SMN associated complexes exist in the neuron. Supporting this hypothesis are electromicroscopy data showing that gold labeling of mSmn signals are found in granules and in locations other than granules in the cytoplasm/axoplasm, indicating different mSmn-associated complexes exist. An axonal-SMN (aSMN) has been reported and is potentially involved in axonogenesis [34]. The aSMN retained the N-terminal region (exons 1–3, encoding the Tudor domain, Gemin2 interacting domain, and self-association domain) of SMN and is thought to have the ability to mediate its axonal functions through interacting with other SMN interacting proteins. We therefore propose that SMN granules from the Golgi network can be transported along the axon. During this process, SMN granules associate with other interacting partners (other SMN-associated complexes or mRNA granules) locally to mediate local axonal regulations.

### Golgi-mediated SMN Granule Secretion Impacts Motor Degeneration in SMA

Disruption of Golgi-mediated granule secretion reduced mSmn levels in neurites (Figure 4B), suggesting that the vesicular transport of mSmn granules may be mediated through the Golgi. Global disruption of granule secretion from the Golgi (including the mSmn granule) causes growth cone defects in NSC34 cells, indicating that proper Golgi function and vesicular transport maintenance is critical to neuronal cells. It was reported that a mutation in the *VAMB* gene, which mediates vesicular trafficking, may contribute to late-onset SMA and amyotrophic lateral sclerosis (ALS) [35]. Additionally, we have previously reported that a microtubule destabilizing protein, stathmin, is aberrantly regulated in an SMA mouse model, has elevated levels in the sciatic axons of SMA-like mice, and causes microtubule defects *in vitro* and *in vivo* [21]. Stathmin is also known to target the Golgi apparatus; in a familial ALS mouse model, stathmin was found to be associated with fragmented Golgi in about 30% of motor neurons [36]. Taken together with our current findings, we propose that unregulated stathmin in SMA associates with a dysfunctional Golgi apparatus, which leads to further reduction of mSmn levels in neurites, contributing to motor neuronal degeneration.

### Conclusions

In the present work, we provide direct evidence showing the involvement of cytoplasmic SMN in the Golgi network. SMN is characteristically carried in a granule from the Golgi and acts like a regulated secretory granule. Disruption of granule secretion from the Golgi resulted in decreased SMN levels in neurites and caused growth cone defects mimicking SMN protein deficiency in neuronal cells. Additionally, Cop-α was shown to regulate SMN granule secretion from the Golgi apparatus. These findings advance our understanding of the characteristics of SMN granules in the cytoplasm and provide a direct link between cytoplasmic SMN granules and the Golgi apparatus, showing Golgi-mediated SMN granule transport.

## Supporting Information

Movie S1 **Colocalization of SMN with β1,4-Gal-T visualized through deconvolution microscopy.** Time series images were taken every 10 sec with *z*-stacks (*z* = 8). Images were constructed into three dimensions and shown 1 plane/sec.
(MOV)

Movie S2 **Colocalization of SMN with SARA-FYVE visualized through deconvolution microscopy.** Time series images were taken every 10 sec with *z*-stacks (*z* = 6). Images were constructed into three dimensions and shown 3 plane/sec.
(MOV)
